# Synthesis, Characterization and Catechol-Based Bioinspired Adhesive Properties in Wet Medium of Poly(2-Hydroxyethyl Methacrylate-*co*-Acrylamide) Hydrogels

**DOI:** 10.3390/polym16020187

**Published:** 2024-01-08

**Authors:** Sebastian Romero-Gilbert, Matías Castro-García, Héctor Díaz-Chamorro, Oscar G. Marambio, Julio Sánchez, Rudy Martin-Trasancos, Matías Inostroza, Claudio García-Herrera, Guadalupe del C. Pizarro

**Affiliations:** 1 Departamento de Química, Facultad de Ciencias Naturales, Matemáticas y Medio Ambiente, Universidad Tecnológica Metropolitana (UTEM), J. P. Alessandri 1242, Santiago 7800002, Chile; sromerog@utem.cl (S.R.-G.); omarambi@utem.cl (O.G.M.); 2 Departamento de Ciencias del Ambiente, Facultad de Química y Biología, Universidad de Santiago de Chile (USACH), Santiago 9170022, Chile; 3 Departamento de Química de los Materiales, Facultad de Química y Biología, Universidad de Santiago de Chile (USACH), Santiago 9170022, Chile; 4 Departamento de Ingeniería Mecánica, Facultad de Ingeniería, Universidad de Santiago de Chile (USACH), Av. Bernardo O’Higgins, Santiago 9170022, Chileclaudio.garcia@usach.cl (C.G.-H.)

**Keywords:** bioinspired adhesive, hydrogels, thermal behavior, mechanical and adhesive properties, swelling behavior

## Abstract

Hydrogels consist of crosslinked hydrophilic polymers from which their mechanical properties can be modulated for a wide variety of applications. In the last decade, many catechol-based bioinspired adhesives have been developed following the strategy of incorporating catechol moieties into polymeric backbones. In this work, in order to further investigate the adhesive properties of hydrogels and their potential advantages, several hydrogels based on poly(2-hydroxyethyl methacrylate-*co*-acrylamide) with *N*′*N*-methylene-bisacrylamide (MBA), without/with L-3,4-dihydroxyphenylalanine (DOPA) as a catecholic crosslinker, were prepared via free radical copolymerization. 2-Hydroxyethyl methacrylate (HEMA) and acrylamide (AAm) were used as comonomers and MBA and DOPA both as crosslinking agents at 0.1, 0.3, and 0.5 mol.-%, respectively. The polymeric hydrogels were characterized by Fourier transform infrared spectroscopy (FT-IR), thermal analysis and swelling behavior analysis. Subsequently, the mechanical properties of hydrogels were determined. The elastic properties of the hydrogels were quantified using Young’s modulus (stress–strain curves). According to the results herein, the hydrogel with a feed monomer ratio of 1:1 at 0.3 mol.-% of MBA and DOPA displayed the highest rigidity and higher failure shear stress (greater adhesive properties). In addition, the fracture lap shear strength of the biomimetic polymeric hydrogel was eight times higher than the initial one (only containing MBA); however at 0.5 mol.-% MBA/DOPA, it was only two times higher. It is understood that when two polymer surfaces are brought into close contact, physical self-bonding (Van der Waals forces) at the interface may occur in an –OH interaction with wet contacting surfaces. The hydrogels with DOPA provided an enhancement in the flexibility compared to unmodified hydrogels, alongside reduced swelling behavior on the biomimetic hydrogels. This approach expands the possible applications of hydrogels as adhesive materials, in wet conditions, within scaffolds that are commonly used as biomaterials in cartilage tissue engineering.

## 1. Introduction

Hydrogels are a class of scaffolds that are commonly used as biomaterials in cartilage tissue engineering. These material types include agarose, alginate, poly(vinyl alcohol) (PVA), poly(ethylene glycol) (PEG), chitosan, fibrin and collagen [1,2], among others.

Hydrogels represent a class of materials with physical (or chemical) crosslinks and high water content. The crosslink process can be ionic or covalent, leading to a solid or gel material by restricting the ability of movement. Crosslinking increases the molecular mass of the polymer, its strength and its mechanical properties and thus its resistance to heat, wear and attack by solvents [3]. On the other hand, the versatility of synthesis and the nature of the precursor allow one to prepare hydrogels with different mechanical and thermal properties. In this way, the mechanical and thermal behavior of the hydrogels can be modulated for specific applications [4], such as materials with adherent, elastic or viscoelastic properties [5,6,7,8]. Therefore, hydrogel materials have become some of the most popular materials depending on their application, such as hair gels and contact lenses [8]; oil recovery; pharmaceutical, agriculture, textile and adherent materials; and water treatment [9,10,11]. Regarding their mechanical properties, during testing, the slope at low strain, also called Young’s modulus or the elastic modulus, is a critical property in understanding the hydrogel’s behavior. In principle, the Young’s modulus of a hydrogel can be measured through the force–deformation (stress–strain) response [4]. The ability of materials to adhere to other materials, providing temporary retention, is called adhesion. The wide-ranging application of adhesive materials implies, scientifically, the necessity to develop hydrophilic polymers with charged and/or nonionic functional groups able to form hydrogen bonds with different surfaces [12]. 

The strength of the hydrogel can be attributed to different types of interactions between the polymer backbone, such as hydrogen bonding [13], electrostatic [14], hydrophobic [15,16] and hydrophilic interactions [17,18]. Moreover, covalently crosslinked hydrogels are not reversible, and, therefore, they display stronger and more stable mechanical properties [19], typified by the elastic modulus. Hydrogels can be crosslinked via thermal polymerization [20,21], photopolymerization [22,23], enzymatic crosslinking [24] and several other methods [25]. 

In terms of swelling behavior and absorption capacity, such properties are the hydrogels’ most advantageous aspects, leading to wide applicative scope. Swelling and absorption properties are attributed to the presence of hydrophilic groups such as –OH, CONH–, –CONH_2_ and –SO_3_H in the network [26]. Therefore, many factors affect the swelling ratio and absorption capacity of hydrogels, such as the network’s structure, the monomer’s composition [27,28], the amount of solvent, the crosslinking degree and the specific stimuli or surrounding medium [29]. Mainly, the hydrophilic/hydrophobic ratio in the polymer backbone affects the swelling behavior and the absorption capacity of hydrogels [30,31]. 

Previous works mention that similar polymer systems exhibit not only pH but also temperature responsivity. Thus, novel brush coatings have been fabricated with glass surface-grafted chains copolymerized using surface-initiated atom transfer radical polymerization (SI-ATRP) from 2-(2-methoxyethoxy)ethyl methacrylate (OEGMA188) and acrylamide (AAm). [32] The thickness of the coatings immersed in water and the morphology of the coatings imaged in air show a temperature response for POEGMA188; however, this response is weak for P(OEGMA188-co-AAm) and absent for PAAm.

In the last decade, many catechol-based bioinspired adhesive materials have been developed by incorporating catechol moieties into well-defined polymeric backbones. Different approaches have been developed to obtain wet adhesive materials based on random copolymers with catechol moieties and, alternatively, on designed polymeric scaffolds incorporating catechol groups [33,34]. For instance, it was found that the adhesion properties increase proportionally to the DOPA content [35,36]. Therefore, the successful design of adhesives based on macromolecular materials with pendant DOPA, or by the extension of catecholic units, would need to achieve a balance between interfacial adhesion and bulk cohesiveness by determining the degree of overall oxidation that affords an optimal mixture of catecholic and o-quinoid moieties [32]. On the other hand, a significant benefit of using hydrogels is their potential as biomaterials that form a scaffold for cartilage repair, and their swelling nature provides an aqueous environment comparable with soft tissue for encapsulated cells [1].

In the present work, we report the synthesis, characterization and applications of catechol-based copolymer hydrogels by using 2-hydroxyethyl methacrylate (HEMA) and acrylamide (AAm) as co-monomers at an equimolar monomer ratio. *N*′*N*-methylene-bisacrylamide (MBA) and L-3,4-dihydroxyphenylalanine (DOPA), at different mol.-%, are used as crosslinkers. Our main goal is to obtain an adhesive material that works in wet conditions. Subsequently, the swelling behavior and absorption capacity of the hydrogels are studied at different pH. The thermal behavior and the mechanical properties are determined and correlated. We evaluate the ability of the materials to adhere to other materials in a wet medium (adhesive properties).

## 2. Experimental Section

### 2.1. Reagents 

2-Hydroxyethyl methacrylate (HEMA, 99 wt. % Merck, Darmstadt, Germany) and acrylamide (AAm, Aldrich CHEMIE, Taufkirchen, Germany) were used as the monomers and ammonium persulfate (APS, 95 wt. %, Merck) as an initiator, all of analytically pure grade. *N*′*N*-methylene-bisacrylamide (MBA, 99 wt. % Merck, Darmstadt, Germany) was used as a crosslinking agent. All chemicals were used as received, without further purification. L-3.4-dihidroxifenilalanina (DOPA, 98 wt. % Sigma-Aldrich, Taufkirchen, Germany) was also used.

### 2.2. Measurement 

#### 2.2.1. Fourier Transform Infrared Spectroscopy (FT-IR)

The IR spectra of the hydrogel samples were measured through a wavenumber range of 500–4000 cm^–1^ with a resolution of 1 cm^–1^ and 128 scans, recorded using a PerkinElmer (St. Louis, MO, USA) FT-IR Spectrometer via the attenuated total reflection method at room temperature. 

#### 2.2.2. Thermal Analysis

The hydrogel sample size (within 3 or 4 ± 0.1 mg), used in each experiment, was analyzed using a thermogravimetric analyzer (TGA/DSC 1, Mettler Toledo, Barcelona, Spain). The measurements were carried out at a heating rate of 10 °C min^−1^ under a nitrogen atmosphere with a flow rate of 150 cm^3^ min^−1^. The thermal analysis, using differential scanning calorimetry (DSC), was carried out in a nitrogen atmosphere with a DSC Mettler Toledo 822^e^ analyzer. The measurements were carried out at a heating rate of 10 °C/min under a nitrogen atmosphere with a flow rate of 50 cm^3^ min^−1^. All experiments were performed in the temperature range from 25 °C to 550 °C in an inert atmosphere (N_2_ gas).

### 2.3. Catechol-Based Bioinspired Adhesives on Poly(HEMA-co-AAm) Hydrogels 

The bioinspired hydrogel was prepared by free radical polymerization in aqueous solution, using HEMA and AAm monomers at a 1:1 molar fraction of monomers and 0.1/0.3 and 0.5 mol.-% of DOPA and 0.1/0.3 and 0.5 mol.-% MBA crosslinker percentage. The polymerization was carried out using 0.5 mol.-% of APS as an initiator in aqueous solution at 70 °C for 2 h, respectively. In accordance with the experimental procedure, a 1:1 feed ratio monomer of HEMA/AAm (15 mmol of each of them) was added in a flask. Then, 0.5 mol.-% of ammonium persulfate (APS) as an initiator, 0.1 mol.-% of MBA and 0.1, 0.3 and 0.5 mol.-% of DOPA were dissolved in 8 mL distilled water at room temperature. After this, the mixture was vacuum-degassed and nitrogen-filled before being heated for 2 h at 70 °C. The poly(HEMA-*co*-Aam) hydrogel functionalized with DOPA was washed with distilled water to remove impurities. Finally, the dried biomimetic hydrogels were characterized using FT-IR, thermal properties, swelling behavior and mechanical properties analysis. The mechanical properties were determined in wet media.

### 2.4. Hydrogel Swelling Studies

#### Effect of Crosslinker MBA/DOPA Composition on Swelling Properties

The swelling properties of the hydrogel samples with different crosslinker percentages (without/with DOPA) were investigated as follows. The key idea was to obtain absorbent polymers with swelling properties suitable for bioinspired applications, where this type of material can be applied in biological areas where low water absorption is needed.

The degree of swelling (*DS*) was calculated using the following equation:

$$DS(\%)=\frac{\left(W_{S}-W_{d}\right)}{W_{d}}\times 100$$

where *W_S_* is the weight of the swollen hydrogel at an equilibrium state, and *W_d_* is the weight of the dried hydrogel (Xerogel). The studies were carried out at 25 °C in buffered solutions, either with 0.01 M citrate buffer (pH = 7) or phosphate buffer (pH = 7). The dried samples of the copolymers were placed in a solution of the defined pH (3, 7 and 10) at 25 °C. In hourly intervals, the sample was removed quickly from the solution.

### 2.5. Mechanical Property Studies

#### 2.5.1. Compression Test

**Figure 1:**
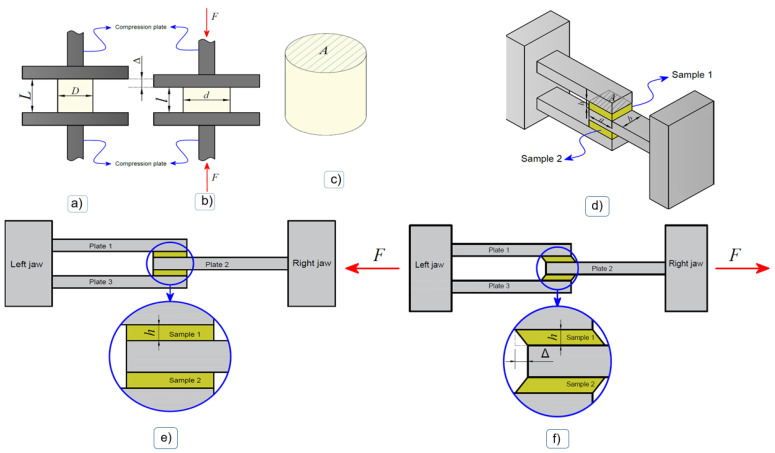
Experimental test scheme. Compression test: (**a**) Mounting of the sample between compression plates, (**b**) deformed configuration, (**c**) sample geometry. A: initial cross-sectional area. Shear test: (**d**) Diagram of the execution of the shear test, (**e**) start of the test, (**f**) deformed configuration during the test [37].

The objective of this test was to obtain the mechanical behavior of the material under uniform compressive stress regimes in wet media. The hydrogel samples were shaped into a cylindrical geometry (see Figure 1a), with initial diameters and heights D = 1 cm and L = 1 cm, respectively, and then subsequently subjected to uniform compression (see diagram in Figure 1b) in an Instron 3342 universal testing machine (Instron, Toronto, ON, Canada), equipped with a 500 N load cell with precision of 0.025 N. The experiment was carried out at room temperature and at a constant displacement rate of 0.7 mm/min, selected to be slow so as to be considered as a quasistatic load (avoiding viscoelastic effects).

In each hydrogel sample, the Cauchy stress was calculated from the force obtained from the load cell during the test and the instantaneous cross-sectional area associated with the instantaneous diameter. The compression strain was calculated from the initial length, the instantaneous length and the compression displacement during the test. When considering an incompressible material, the instantaneous cross-sectional area can be estimated as being the initial cross-sectional area (undeformed) associated with the diameter; see Figure 1a,b.

After the test was performed, stress–strain was computed, and the initial slope (at low strains) was obtained. 

#### 2.5.2. Shear Test

The objective of this test was to subject samples of the material to a state of shear stress. For this purpose, two portions of hydrogel were positioned between two cavities formed by three plates of the assembly accessory shown in Figure 1. In an idealized way, each sample adopted a parallelepiped geometry with specific dimensions (see Figure 1c). Then, the samples were tested in a Cellscale 5000 biaxial tensile testing machine, (Cellscale Biomaterials Testing, Waterloo, ON, Canada), equipped with a 10 N load cell, through relative motion between the plates in the longitudinal direction of the assembly (Figure 1d–f), at a constant displacement rate of 0.7 mm/min.

The shear stress in the reference configuration was calculated as the shear force in one of the samples, which, given the double shear configuration, could be calculated corresponding to the initial cut area (see Figure 1c). Furthermore, the shear deformation was approximated corresponding to the relative displacement between jaws during the test and the distance between one and another plate (see Figure 1d–f). 

After the test, the initial slope (low strains) was obtained from the shear stress–strain curve.

## 3. Results and Discussion

### 3.1. Synthesis and Characterization by FT-IR, TGA and DSC 

The broad range of applications of biomimetic hydrogels is based on their versatility in terms of their chemical structures as well as their physical properties. The nature of the monomers and the experimental method used for the synthesis allow one to obtain hydrogels with different thermal and mechanical properties. The synthesis conditions and yields of the poly(HEMA-*co*-AAm) hydrogels produced in this work are shown in Table 1. Accordingly, the hydrogels were always prepared with yields higher than 90%.

### 3.2. FT-IR Spectroscopic Studies

The hydrogels were characterized by FT-IR spectroscopy, as depicted in Table 2. The FT-IR of the poly(HEMA-*co*-AAm) hydrogels prepared in the absence and presence of DOPA at a 1:1 monomer ratio, at 0.1, 0.3 and 0.5 mol.-% of MBA, shows the typical bands of both comonomers. Table 2 shows the typical signals of both comonomers, with slight variations in the intensity of the carbonyl bands from amide and ester, approximately at 1660 and 1715 cm^−1^, respectively. Among the most characteristic bands, the FT-IR spectrum shows bands in the range of 3340–3448 cm^−1^, attributed to the stretching of the hydroxyl (–OH) groups, and at 3000–2835 cm^−1^, related to the C-H stretching of methyl (–CH_3_), methylene (–CH_2_) and methyne (>CH-). The band located approximately at 1715 cm^−1^ is related to the >C=O stretching of the ester from HEMA and the ones recorded at 1611–1600 and 1437–1450 cm^−1^ arise from NH_2_CO (C-O and N-H, respectively) deformation of the amide group [38].

The biomimetic hydrogel was prepared with P(HEMA-*co*-AAm) at a 1:1 feed monomer ratio (HEMA/AAm), with MBA and L-3,4-dihydroxyphenylalanine (DOPA) as catecholic crosslinkers. The FT-IR spectra of the hydrogels showed the characteristic bands of both monomers. In the spectra, changes were observed in the signal intensity due to the presence of the crosslinker agents (MBA/DOPA). This outcome could be explained by considering the changes in the individual intensities of the peaks corresponding to carbonyl groups, probably due to the small content of the MBA/DOPA agents. 

On the other hand, the FT-IR spectra of the hydrogel with DOPA show the stretching and bending of vibrational modes from –OH at 3345 cm^–1^ approximately. Moreover, the bands at 1072 and 1153 cm^–1^, resembling the phenolic -COH band, were also recorded according to the literature [39]. Additionally, the C=C stretching of the aromatic from catechol and the –COO stretching were identified between 895 and 680 cm^–1^ [39,40,41]; see Table 2.

### 3.3. Thermal Behavior by TGA and DSC

#### 3.3.1. Thermal Analysis for Copolymer with MBA without/with DOPA by TGA

**Figure 2:**
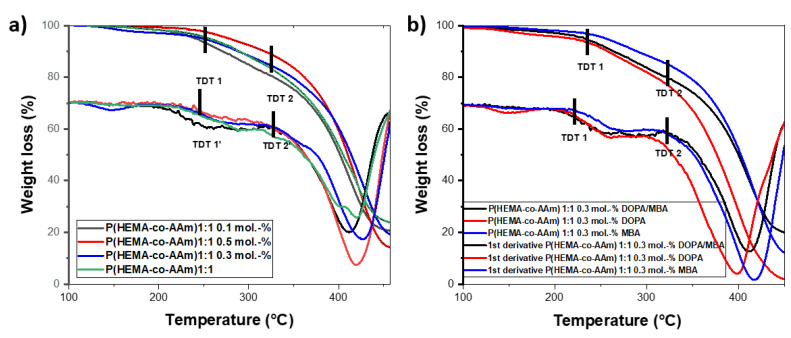
Thermograms and their first derivatives of P(HEMA-co-AAm): at 1:1 feed monomer ratio (**a**) with different mol.-% MBA crosslinking agent and (**b**) with 0.3 mol.-% crosslinking agent and DOPA.

The copolymers and hydrogels at 1:1 feed ratio monomers, without or with DOPA, show a first thermogramatic step of eliminating water/monomer residues, above which the thermograms present two-step degradations; see Figure 2a,b respectively. 

The hydrogels with a 1:1 feed monomer ratio at different MBA percentages (0.1, 0.3 and 0.5 mol.-%) presented similar thermal behaviors in terms of two-step degradation, with initially extrapolated thermal decomposition temperatures (TDT_1_) at 254.9 °C, 261.8 °C and 268.6 °C, respectively, and a second-step degradation (TDT_2_) at 411.7 °C, 421.6 °C and 429.2 °C, respectively. As can be noted, for higher MBA content, the greater thermal stability of the polymer is observed. On the other hand, the hydrogels at 0.3 mol.-% MBA/DOPA presented similar thermal behavior to the hydrogel with 0.3 mol.-% MBA, but the hydrogel with 0.3 mol.-% DOPA exhibited lower thermal stability (see Figure 2b). In the same way, two-step degradation was observed for the hydrogels containing 0.3 mol.-% of DOPA. The greatest weight loss occurred between 350 and 450 °C, with a percentage of residual mass ranging from 16 to 20% for the different hydrogels. The lowest residual mass was 20.09%, for the feed ratio of 1:1 at 0.3 mol.-% of MBA and DOPA; see Figure 2b.

#### 3.3.2. Thermal Analysis of Hydrogels by DSC

For crosslinked polymers, the glass transition temperature (Tg) is influenced by the presence of a reticulation reaction [42]. The effect of the MBA crosslinking reaction within the P(HEMA-*co*-AAm) hydrogels could be investigated based on the increase or decrease in the Tg [43]. In all cases, the glass transition temperatures (Tg) were recorded between 100 and 118 °C; these Tg values are lower than that for lineal polymer chains [44]. It is important to emphasize that the chemical nature of the polymer is primarily responsible for changes in the Tg value. Results obtained from the DSC curve showed the formation of amorphous polymers with the presence of Tg after the second heating, once the thermal history of the material had been erased. The DSC curves of P(HEMA-*co*-AAm) at a 1:1 feed ratio of monomers with and without MBA, and with DOPA, were obtained; see Table 3. The catechol-meditated crosslinking provided an enhancement in the Tg temperature. The DSC curves of the P(HEMA-*co*-AAm) hydrogels showed that the Tg of the hydrogel with 0.1 mol.-% of DOPA and MBA was higher than the Tg of the hydrogel with MBA alone (without DOPA). This result suggests that the addition of MBA and DOPA increases the crosslinking. In general, the hydrogel with MBA shows a lower Tg than that with DOPA, increasing for 0.5 mol.-%, respectively. This could indicate that the particular monomer moiety from HEMA, with an –OH functional group, also contributes to a certain degree of physical interaction with the DOPA agent, due mainly to the formation of hydrogen bonds, leading to the formation of greater stiffness in the hydrogel, causing a higher Tg value; see Table 3.

**Figure 3:**
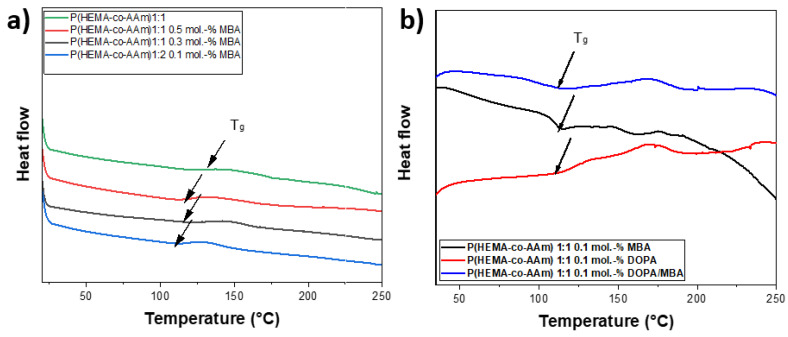
(**a**) DSC curves of P(HEMA-*co*-AAm) at 1:1 feed monomer ratio at different percentages of MBA (0.1, 0.3 and 0.5.-mol.%); (**b**) DSC curves of P(HEMA-*co*-AAm) at feed monomer ratio of 1:1 at 0.5.-mol.% of MBA, 0.5.-mol.% of DOPA and 0.5.-mol.% of DOPA/MBA.

In general, the Tg of the hydrogel at 1:1 increased as the mol.-% of MBA increased, suggesting that the increase in the percentage of MBA was proportional to the crosslinking content; see Figure 3. 

### 3.4. Mechanical Properties

**Figure 4:**
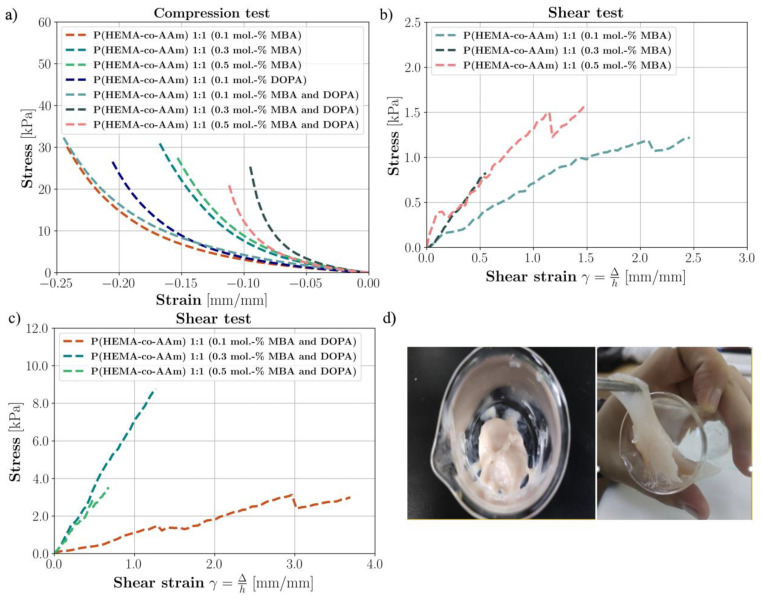
(**a**) The compression test for P(HEMA-co-AAm) hydrogels at 1:1 feed monomer ratio at 0.1, 0.3 and 0.5 mol.-% MBA with/without DOPA crosslinker. (**b**) Lap shear strength testing of P(HEMA-co-AAm) hydrogels at 1:1 feed monomer ratio for 0.1, 0.3 and 0.5 mol.-% MBA with/without DOPA crosslinker. (**c**) Lap shear strength testing of P(HEMA-co-AAm) hydrogels at 1:1 feed monomer ratio for 0.1, 0.3 and 0.5 mol.-% MBA. (**d**) Image of the hydrogel containing MBA and DOPA.

The mean compressive and shear stress–strain curves of the hydrogels are shown in Figure 4a–c. Their mechanical properties are summarized in Table 4. 

In general, it is reported that as the crosslinking density of an elastic material increases, the compressive strength increases and the Young’s modulus at break decreases [45]. In this work, the mechanical behavior of the poly(HEMA-*co*-AAm) hydrogels immersed in water at different feed monomer ratios and percentages of MBA (mol.-%) were studied and are presented as follows (Table 4).

#### 3.4.1. Biomimetic Hydrogels as Adhesive Materials with Improved Mechanical Properties in Wet Conditions

Table 4 shows the values of the compression test applied to various series of wet hydrogel samples (with sample volumes of approximately 1 cm^3^). The samples varied in their initial crosslinking percentages at 0.1, 0.3 and 0.5 mol.-% of MBA, respectively, at a monomer ratio of 1:1. It is important to highlight that most of the samples did not exhibit a clear failure point; thus, the mechanical properties are referenced to their elastic modulus. Moreover, Figure 4a shows the stress–strain curves obtained from the experimentation, where the minimum strain of each sample is the strain in which the force had reached 5 N.

The hydrogels at a feed ratio composition of 1:1 and 0.1 mol.-% of MBA presented an average modulus of 30.42 kPa (±3.43, n = 3), indicating a minor stiffness modulation in compression compared to the other hydrogels with/without DOPA. As the results are respective to the mechanical properties, as the mol.-% of MBA content increased from 0.1 to 0.5 mol.-%, the Young’s modulus of the hydrogels increased from 30.42 to 56.06 kPa, (±4.38, n = 5). This result is significant, as it shows that the Young’s modulus increases, indicating a stiffer material. In this way, this could indicate that the 1:1 initial monomer composition (AAm/HEMA) generates a certain degree of physical crosslinking through Van der Waals interactions and hydrogen bonds between the amide groups, giving the hydrogel more flexibility and resistance to deformation, which indicates that this hydrogel could have greater viscoelastic properties; see Figure 4a.

#### 3.4.2. Effect of MBA and DOPA Crosslinker Agents at 0.1, 0.3 and 0.5 mol.-% on the Mechanical Properties of the Biomimetic Hydrogels at 1:1 Feed Monomer Ratio

Table 4 exhibits the mechanical properties of the biomimetic hydrogels at a 1:1 feed monomer ratio, at 0.1, 0.3 and 0.5 mol.-% of MBA and 0.1, 0.3 and 0.5 mol.-% MBA/DOPA (as catecholic crosslinkers); see Figure 4a. In general, it was observed that with the DOPA addition, the crosslinking density increased and thus the Young’s modulus increased. For example, at 0.5 mol.-% of MBA and DOPA, an average modulus value of 58.86 kPa (±2.04, n = 3) was observed, corresponding to higher stiffness. The introduction of DOPA resulted in slightly better mechanical properties under humid conditions compared to the hydrogel synthesized with MBA alone. In brief, the hydrogel at a 1:1 feed monomer ratio at 0.5 mol.-% of MBA and DOPA had greater viscoelastic properties. The stiffness of the hydrogel with only MBA was lower than that of the hydrogel with MBA/DOPA at the same percentage of MBA and that with only DOPA, in wet conditions, in all cases. 

#### 3.4.3. Shear Test between the Biomimetic Polymeric Hydrogel and a Rigid Substrate

A shear test to verify the adhesion between a polymeric hydrogel and a rigid substrate was performed in this work. Lap shear strength testing measures the ability of a material to withstand stresses set in a plane, where the exerted shear force moves the two substrates in opposite directions; see Figure 1. Table 5 exhibits the ability of the materials to adhere to other materials, providing temporary retention (adhesive testing), for the biomimetic hydrogels at a 1:1 feed monomer ratio and at 0.1, 0.3 and 0.5 mol.-% of MBA and 0.1, 0.3 and 0.5 mol.-% MBA/DOPA (as catecholic crosslinker); see Figure 4b–d.

Using the results, it can be observed that with the addition of the DOPA crosslinker agent to the hydrogel, the crosslinking density increases. Both the shear stress failure values and shear moduli of elasticity increase for a feed monomer ratio of 1:1 at 0.1, 0.3 and 0.5 mol.-% of MBA and DOPA, respectively. The introduction of DOPA resulted in the improved mechanical properties in wet conditions of the hydrogels with respect to those only with MBA. Furthermore, the hydrogel with a feed monomer ratio of 1:1 at 0.1 mol.-% MBA and DOPA displayed higher flexibility and lower resistance; however, the hydrogel at 0.3 mol.-% MBA and DOPA sustained higher tension at shear failure, i.e., it had greater shear resistance against the applied force (Figure 4b–d). It was observed that with the addition of DOPA (from 0.1 to 0.3 mol.-%) in wet conditions, the shear stress failure increased (improved adhesive properties). According to these results, it can be suggested that when two polymer surfaces are brought into close contact, physical self-bonding at the interface may be expected in the –OH interaction with the wet contacting surfaces [46].

In brief, the hydrogel with a feed monomer ratio of 1:1 at 0.3 mol.-% of MBA and DOPA displayed the highest rigidity and higher shear stress failure (greater adhesive properties). It was observed that the fracture lap shear strength of the biomimetic polymeric hydrogel was eight times higher than the initial one only with MBA at a concentration of 0.3 mol.-% MBA/DOPA, and the result for that at 0.5 mol.-% MBA/DOPA was two times higher than for the hydrogel only with MBA (see Figure 4b–d). For this to occur, chain interpenetration across the interface needs to take place, since the forces of physical attraction (Van der Waals forces) between the molecular groups of the chains located in the outermost layers of the contacting surfaces are not sufficient to give rise to effective interface resistance to the mechanical load [46]. In addition, it is seen from the literature that the intrinsic mechanical properties of agarose hydrogels, commonly used for cartilage tissue engineering studies, are reported to depend on their concentration [47,48]. Here, the average ramp modulus of a 2% agarose gel in tension was 24.9 kPa, compared with 55.6 kPa in compression. The average tensile equilibrium modulus was 39.7 kPa, significantly higher than the compressive equilibrium modulus of 14.2 kPa [49]. These results have great significance for the interpretation of our results regarding potential hydrogel applications in biomaterials. Therefore, many scientists and engineers are interested in materials that can be used to develop skin-like electronics or biomaterials. To imitate the soft and elastic nature of skin, functional materials need to be elastic (0.4–1.9 MPa) and flexible (elongation higher than 180%) [50].

### 3.5. Swelling Studies 

#### 3.5.1. Influence of the pH on Hydration Capacity of the Hydrogels

**Figure 5:**
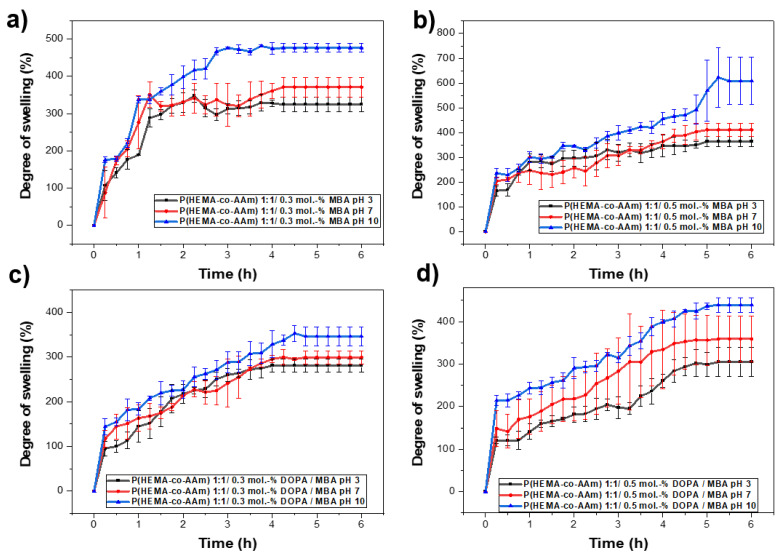
(**a**) Influence of the pH on hydration capacity at pH 3, 7 and 10 for 1:1 feed monomer ratio at (**a**) 0.3 mol.-% MBA, (**b**) 0.5 mol.-% MBA, (**c**) 0.3 mol.-% MBA /DOPA, (**d**) 0.5 mol.-% MBA/DOPA.

The swelling behavior of the poly(HEMA-*co*-AAm) hydrogels was investigated as a function of pH via the immersion of the gels in buffered solutions at pH = 3, 7 and 10 at room temperature (25 °C). Figure 5 shows the swelling behavior of the hydrogels after different swelling times. The swelling of the hydrogels increased in the pH range of 7–10, where the maximum swelling could be observed. This could be attributed to the complete interaction of the functional groups with the water at this pH range. Moreover, the swelling increased with time; however, after some time, it leveled off. This value of swelling can be referred to as the equilibrium swelling percentage.

#### 3.5.2. Influence of the Percentage of MBA with or without DOPA at 1:1 Feed Monomer Ratio on Hydration Capacity at Different pH 

For the same initial composition of monomers in the copolymer, keeping the percentage of crosslinking agent constant and varying the pH from 3 and 5 to 10, it can be observed that the highest percentage of hydration is obtained as the pH increases; see Table 6 and Figure 5a,b. Subsequently, by increasing the percentage of the crosslinking agent and maintaining the initial composition of monomers in the copolymer and the pH, it was possible to observe a decrease in the percentage of hydration; see Table 6 and Figure 5a,b. On the other hand, a higher percentage of hydration is always observed for the lower percentage of MBA/DOPA crosslinking agents (at 0.1 mol.-% MBA). Thus, the behavior of the crosslinked hydrogel with a feed monomer ratio of 1:1 at 0.1, 0.3 and 0.5 mol.-% DOPA and MBA also was analyzed. Figure 5b shows the behavior of the copolymer at different pH levels (3, 7, 10) and at 0.1 mol.-% of MBA and DOPA.

Regarding the hydration percentages, the hydrogel with MBA and with/without DOPA crosslinker agents (at 0.1, 0.3 and 0.5 mol.-%) presented an increase in the hydration percentage as the pH increased from 3 to 10. However, in the hydrogel only with the MBA crosslinker, a higher percentage of hydration was observed at the same pH (up to 1806% at pH 10) compared with the hydrogel with DOPA (up to 800% at pH 10); see Table 6. In this study, in response to the different hydration percentages observed at different pH levels, a potential mechanism that influences the hydration capacity is suggested, where hydrogen bonds play a leading role between the functional groups of HEMA and AAm, which include free hydroxyl and amide groups. These functional groups interact with the hydrogens of water in buffer solutions at different pH levels, exhibiting different behavior in wettability for pH = 3, 7 and 10, favoring these interactions at a more basic pH. On the other hand, lower percentages of hydration are observed when DOPA is incorporated into the system. This result is attributed to the hydrogen bonds generated between the functional groups, where DOPA also participates in the three-dimensional network, which could block the monomers’ functional groups, providing less space in the cells of the three-dimensional macrostructure. With fewer free functional groups to generate these Van der Waals forces with water molecules, this affects the ability to absorb and retain them in the three-dimensional network.

## 4. Conclusions

All polymeric hydrogels are insoluble in water and in some common organic solvents. Here, an experimental procedure was designed to improve the mechanical properties of P(HEMA-co-AAm) hydrogels in wet conditions and minimize the swelling behavior due to water adsorption by catechol-meditated crosslinking. The biomimetic P(HEMA-co-AAm) hydrogels were prepared from both monomers (HEMA and AAm) with MBA as a crosslinker, mixing them with L-3,4-dihydroxyphenylalanine (DOPA) as a catecholic crosslinker. For the hydrogel at a 1:1 feed monomer ratio at 0.1, 0.3 and 0.5 mol.-% of MBA and DOPA, the catechol-meditated crosslinking provided an enhancement in the Tg temperature and stiffness in wet conditions, compared with the hydrogel only with the MBA crosslinker agent. On the other hand, with respect to thermal stability, the hydrogels with a 1:1 feed ratio at 0.5 mol.-% MBA presented similar behavior to the hydrogels with 0.5 mol.-% DOPA, with two step degradation; however, in the final instance, the hydrogel with both MBA and DOPA crosslinkers exhibited higher thermal stability. Poly(HEMA-co-AAm) hydrogels with an MBA mol.-% crosslinking agent with/without DOPA were prepared by free radical copolymerization in aqueous solutions. With respect to the mechanical properties, as the mol.-% of MBA content increased from 0.1 to 0.5 mol.-% at a 1:1 feed monomer ratio, the compressive strength of the hydrogels increased significantly. The introduction of DOPA resulted in an improvement in the failure shear stress value, shear modulus and elasticity properties, increasing for a feed monomer ratio of 1:1 with MBA and DOPA (of 0.1, 0.3 and 0.5 mol.-%), respectively. 

The swelling characteristics of the copolymer gels showed that the catechol-meditated crosslinking reduced the swelling behavior of the hydrogels. This strategy expands the possible applications for the use of biomimetic hydrogel materials as biomaterials and adhesive materials in wet conditions. The catechol-meditated crosslinking provided an enhancement in stiffness in wet conditions compared to the pure hydrogels, and it reduced the swelling behavior of the hydrogels. This approach expands the possible applications for hydrogels as adhesive biomaterials in wet conditions. 

## Figures and Tables

**Table 1:** Experimental conditions of the synthesis of bioinspired hydrogels with 0.1, 0.3 and 0.5 mol.-% MBA and DOPA in 8 mL of water and 0.5 mol.-% of APS.

Polymer Sample	Feed Monomer Ratio	HEMA (mol/g)	AAm (mol/g)	MBA (mol.-%)	DOPA	Yield (%)
P(HEMA-*co*-AAm) 1:1	1:1	0.015/1.950	0.015/1.065	Without	without	95
P(HEMA-*co*-AAm) 1:1/0.1% MBA	1:1	0.015/1.950	0.015/1.065	0.1	without	90
P(HEMA-*co*-AAm) 1:1/0.3% MBA	1:1	0.015/1.950	0.015/1.065	0.3	without	92
P(HEMA-*co*-AAm) 1:1/0.5% MBA	1:1	0.015/1.950	0.015/1.065	0.5	without	92
P(HEMA-*co*-AAm) 1:1/0.1% MBA/0.1% DOPA	1:1	0.015/1.950	0.015/1.065	0.1	0.1	90
P(HEMA-*co*-AAm) 1:1/0.3% MBA/0.3% DOPA	1:1	0.015/1.950	0.015/1.065	0.3	0.3	90
P(HEMA-*co*-AAm) 1:1/0.5% MBA/0.5% DOPA	1:1	0.015/1.950	0.015/1.065	0.5	0.5	90

APS: 0.019 M, 0.5 mol.-% (0.0348 g); *N*′*N*-methylene-bisacrylamide (MBA), L-3,4-dihydroxyphenyl alanine (DOPA) as catecholic crosslinker.

**Table 2:** Characteristic IR absorption bands of the polymeric systems poly(HEMA-*co*-AAm).

System	Vibration Bands [ν (cm^−1^)]					
	Stretching					
	O-H: -OH	C-H: >CH, -CH_2_-, -CH_3_	C-O: -CO_2_R	C-O: H_2_NCO-	N-H: H_2_NCO-	C-H: >CH-, -CH_2_-, -CH_3_
P(HEMA-co-AAm) 1:1	3343	3000-2867	1717	1660	1610	1446
P(HEMA-co-AAm) 1:1/0.1 mol.-% MBA	3340	3000-2865	1716	1660	1607	1460
P(HEMA-co-AAm) 1:1/0.3 mol.-% MBA	3347	3000-2860	1713	1665	1600	1453
P(HEMA-co-AAm) 1:1/0.5 mol.-% MBA	3440	3000-2857	1714	1660	1608	1437
P(HEMA-co-AAm) 1:1/0.5 mol.- % DOPA	3348	2982-2835	1715	1662	1607	1447
P(HEMA-co-AAm) 1:1/0.5 mol.-% MBA/DOPA	3445	3000-2854	1714	1662	1611	1450

**Table 3:** Glass transition temperatures (Tg) of hydrogels with and without crosslinker agent (MBA).

Hydrogel 1:1 0.0 mol.-% MBA 127.4	Glass Transition Temperature (Tg, °C)		
	0.1 mol.-% MBA	0.3 mol.-% MBA	0.5 mol.-% MBA
	100.5	112.6	115.2
P(HEMA-co-AAm) 1:1	0.1 mol.-% MBA/DOPA	0.3 mol.-% MBA/DOPA	0.5 mol.-% MBA/DOPA
	108.3	115.6	118.5

**Table 4:** Compression test for P(HEMA-co-AAm) hydrogels: effect of the different mol.-% of MBA and DOPA at 1:1 feed monomer ratio on mechanical properties of the hydrogels.

Hydrogel Samples	Young’s Modulus (E) (kPa)
P(HEMA-co-AAm) 1:1 (MBA 0.1 mol.-% MBA)	30.42 ± 3.43
P(HEMA-co-AAm) 1:1 (MBA 0.3 mol.-% MBA)	50.80 ± 1.57
P(HEMA-co-AAm) 1:1 (MBA 0.5 mol.-% MBA)	56.05 ± 4.38
P(HEMA-co-AAm) 1:1 (0.1 mol.-% DOPA)	33.77 ± 2.74
P(HEMA-co-AAm) 1:1 (0.1 mol.-% MBA and DOPA)	48.27 ± 1.71
P(HEMA-co-AAm) 1:1 (0.3 mol.-% MBA and DOPA)	54.63 ± 3.14
P(HEMA-co-AAm) 1:1 (0.5 mol.-% MBA and DOPA)	58.86 ± 2.04

**Table 5:** Adhesion test: effect of MBA and DOPA crosslinker agents at 0.1 and 0.3 mol.-% on the mechanical properties of the biomimetic poly(HEMA-co-AAm) hydrogels at 1:1 feed ratio monomer ratio.

Sample	Failure Shear Stress (kPa)	Shear Modulus of Elasticity (kPa)
P(HEMA-co-AAm) 1:1 /0.1 mol.-% MBA	1.37 ± 0.07	0.58 ± 0.10
P(HEMA-co-AAm) 1:1 /0.3 mol.-% MBA	1.19 ± 0.16	1.58 ± 0.36
P(HEMA-co-AAm) 1:1 0.5 mol.-% MBA	1.88 ± 0.17	1.14 ± 0.27
P(HEMA-co-AAm) 1:1 (0.1 mol.-% MBA/0.1 mol.-% DOPA)	3.58 ± 0.94	0.97 ± 0.32
P(HEMA-co-AAm) 1:1 (0.3 mol.-% MBA/0.3 mol.-% DOPA)	9.67 ± 1.55	7.11 ± 1.83
P(HEMA-co-AAm) 1:1 (0.5 mol.-% MBA/0.5 mol.-% DOPA)	4.07 ± 0.44	5.18 ± 0.56

**Table 6:** Influence of crosslinking percentage of MBA/DOPA on hydration capacity at different pH.

Sample	Feed Monomer Ratio	Water Absorbency Capacity g/g at 5 h of Hydration					
		0.1 mol.-% MBA		0.3 mol.-% MBA		0.5 mol.-% MBA	
		pH	(%)	pH	(%)	pH	(%)
P(HEMA-*co*-AAm)	1:1	pH 3	938 ± 21	pH 3	364 ± 20	pH 3	324 ± 20
P(HEMA-*co*-AAm)	1:1	pH 7	1392 ± 30	pH 7	411 ± 25	pH 7	371 ± 26
P(HEMA-*co*-AAm)	1:1	pH 10	1806 ± 36	pH 10	608 ± 95	pH 10	476 ± 12
		0.1 mol.-% MBA/DOPA		0.3 mol.-% MBA/DOPA		0.5 mol.-% MBA/DOPA	
		pH	(%)	pH	(%)	pH	(%)
P(HEMA-*co*-AAm)	1:1	pH 3	408 ± 12	pH 3	280 ± 15	pH 3	305 ± 34
P(HEMA-*co*-AAm)	1:1	pH 7	709 ± 63	pH 7	299 ± 14	pH 7	359 ± 54
P(HEMA-*co*-AAm)	1:1	pH 10	824 ± 21	pH 10	346 ± 21	pH 10	439 ± 17

## Data Availability

Data are contained within the article.

## References

[1] Lum L., Elisseeff J. (2003). Injectable Hydrogels for Cartilage Tissue Engineering. Top. Tissue Eng..

[2] Elisseeff J., Puleo C., Yang F., Sharma B. (2005). Advances in Skeletal Tissue Engineering with Hydrogels. Orthod. Craniofacial Res..

[3] Maitra J., Shukla V.K. (2014). Cross-Linking in Hydrogels—A Review. Am. J. Polym. Sci..

[4] Stojkov G., Niyazov Z., Picchioni F., Bose R.K. (2021). Relationship between Structure and Rheology of Hydrogels for Various Applications. Gels.

[5] Anseth K.S., Bowman C.N., Brannon-Peppas L. (1996). Mechanical Properties of Hydrogels and Their Experimental Determination. Biomaterials.

[6] Ahearne M., Yang Y., Liu K. (2008). Mechanical Characterisation of Hydrogels for Tissue Engineering Applications. Tissue Eng..

[7] Oyen M.L. (2014). Mechanical Characterisation of Hydrogel Materials. Int. Mater. Rev..

[8] Vedadghavami A., Minooei F., Mohammadi M.H., Khetani S., Rezaei Kolahchi A., Mashayekhan S., Sanati-Nezhad A. (2017). Manufacturing of Hydrogel Biomaterials with Controlled Mechanical Properties for Tissue Engineering Applications. Acta Biomater..

[9] Sangeetha N.M., Maitra U. (2005). Supramolecular Gels: Functions and Uses. Chem. Soc. Rev..

[10] Zhang J., Wang A. (2007). Study on Superabsorbent Composites. IX: Synthesis, Characterization and Swelling Behaviors of Polyacrylamide/Clay Composites Based on Various Clays. React. Funct. Polym..

[11] Wu L., Liu M., Liang R. (2008). Preparation and Properties of a Double-Coated Slow-Release NPK Compound Fertilizer with Superabsorbent and Water-Retention. Bioresour. Technol..

[12] Khutoryanskiy V.V. (2011). Advances in Mucoadhesion and Mucoadhesive Polymers. Macromol. Biosci..

[13] Wang Y.J., Zhang X.N., Song Y., Zhao Y., Chen L., Su F., Li L., Wu Z.L., Zheng Q. (2019). Ultrastiff and Tough Supramolecular Hydrogels with a Dense and Robust Hydrogen Bond Network. Chem. Mater..

[14] Liang Y., Xue J., Du B., Nie J. (2019). Ultrastiff, Tough, and Healable Ionic-Hydrogen Bond Cross-Linked Hydrogels and Their Uses as Building Blocks to Construct Complex Hydrogel Structures. ACS Appl. Mater. Interfaces.

[15] Zhang X.N., Wang Y.J., Sun S., Hou L., Wu P., Wu Z.L., Zheng Q. (2018). A Tough and Stiff Hydrogel with Tunable Water Content and Mechanical Properties Based on the Synergistic Effect of Hydrogen Bonding and Hydrophobic Interaction. Macromolecules.

[16] Chang X., Geng Y., Cao H., Zhou J., Tian Y., Shan G., Bao Y., Wu Z.L., Pan P. (2018). Dual-Crosslink Physical Hydrogels with High Toughness Based on Synergistic Hydrogen Bonding and Hydrophobic Interactions. Macromol. Rapid Commun..

[17] Mredha M.T.I., Pathak S.K., Tran V.T., Cui J., Jeon I. (2019). Hydrogels with Superior Mechanical Properties from the Synergistic Effect in Hydrophobic–Hydrophilic Copolymers. Chem. Eng. J..

[18] Oveissi F., Naficy S., Le T.Y.L., Fletcher D.F., Dehghani F. (2018). Tough and Processable Hydrogels Based on Lignin and Hydrophilic Polyurethane. ACS Appl. Bio Mater..

[19] Chang B., Ahuja N., Ma C., Liu X. (2017). Injectable Scaffolds: Preparation and Application in Dental and Craniofacial Regeneration. Mater. Sci. Eng. R Rep..

[20] Wang H., Zhu D., Paul A., Cai L., Enejder A., Yang F., Heilshorn S.C. (2017). Covalently Adaptable Elastin-Like Protein–Hyaluronic Acid (ELP–HA) Hybrid Hydrogels with Secondary Thermoresponsive Crosslinking for Injectable Stem Cell Delivery. Adv. Funct. Mater..

[21] Shin J.Y., Yeo Y.H., Jeong J.E., Park S.A., Park W.H. (2020). Dual-Crosslinked Methylcellulose Hydrogels for 3D Bioprinting Applications. Carbohydr. Polym..

[22] Brown T.E., Carberry B.J., Worrell B.T., Dudaryeva O.Y., McBride M.K., Bowman C.N., Anseth K.S. (2018). Photopolymerized Dynamic Hydrogels with Tunable Viscoelastic Properties through Thioester Exchange. Biomaterials.

[23] Zhou Y., Liang K., Zhao S., Zhang C., Li J., Yang H., Liu X., Yin X., Chen D., Xu W. (2018). Photopolymerized Maleilated Chitosan/Methacrylated Silk Fibroin Micro/Nanocomposite Hydrogels as Potential Scaffolds for Cartilage Tissue Engineering. Int. J. Biol. Macromol..

[24] Kim S.H., Kim K., Kim B.S., An Y.H., Lee U.J., Lee S.H., Kim S.L., Kim B.G., Hwang N.S. (2020). Fabrication of Polyphenol-Incorporated Anti-Inflammatory Hydrogel via High-Affinity Enzymatic Crosslinking for Wet Tissue Adhesion. Biomaterials.

[25] Wei Q., Duan J., Ma G., Zhang W., Wang Q., Hu Z. (2019). Enzymatic Crosslinking to Fabricate Antioxidant Peptide-Based Supramolecular Hydrogel for Improving Cutaneous Wound Healing. J. Mater. Chem. B.

[26] Hamidi M., Azadi A., Rafiei P. (2008). Hydrogel Nanoparticles in Drug Delivery. Adv. Drug Deliv. Rev..

[27] Guilherme M.R., Reis A.V., Takahashi S.H., Rubira A.F., Feitosa J.P.A., Muniz E.C. (2005). Synthesis of a Novel Superabsorbent Hydrogel by Copolymerization of Acrylamide and Cashew Gum Modified with Glycidyl Methacrylate. Carbohydr. Polym..

[28] Patachia S., Valente A.J.M., Baciu C. (2007). Effect of Non-Associated Electrolyte Solutions on the Behaviour of Poly(Vinyl Alcohol)-Based Hydrogels. Eur. Polym. J..

[29] Peppas N.A., Bures P., Leobandung W., Ichikawa H. (2000). Hydrogels in Pharmaceutical Formulations. Eur. J. Pharm. Biopharm..

[30] Xiong Z.C., He C.C., Huang X., Xu L., Zhang L.F., Xiong C. (2007). Preparation and Properties of Thermo-Sensitive Hydrogels of Konjac Glucomannan Grafted n-Isopropylacrylamide for Controlled Drug Delivery. Iran. J. Polym. Sci. Technol..

[31] Alvarez-Lorenzo C., Concheiro A. (2002). Reversible Adsorption by a PH- and Temperature-Sensitive Acrylic Hydrogel. J. Control. Release.

[32] Shymborska Y., Stetsyshyn Y., Awsiuk K., Raczkowska J., Bernasik A., Janiszewska N., Da̧bczyński P., Kostruba A., Budkowski A. (2023). Temperature- and pH-Responsive Schizophrenic Copolymer Brush Coatings with Enhanced Temperature Response in Pure Water. ACS Appl. Mater. Interfaces.

[33] Westwood G., Horton T.N., Wilker J.J. (2007). Simplified Polymer Mimics of Cross-Linking Adhesive Proteins. Macromolecules.

[34] Lee B.P., Dalsin J.L., Messersmith P.B. (2002). Synthesis and Gelation of DOPA-Modified Poly(Ethylene Glycol) Hydrogels. Biomacromolecules.

[35] Lee B.P., Chao C.Y., Nelson Nunalee F., Motan E., Shull K.R., Messersmith P.B. (2006). Rapid Gel Formation and Adhesion in Photocurable and Biodegradable Block Copolymers with High DOPA Content. Macromolecules.

[36] Anderson T.H., Yu J., Estrada A., Hammer M.U., Waite J.H., Israelachvili J.N. (2010). The Contribution of DOPA to Substrate-Peptide Adhesion and Internal Cohesion of Mussel-Inspired Synthetic Peptide Films. Adv. Funct. Mater..

[37] Núñez C., Celentano D. (2005). Caracterización Experimental y Numérica de Compuestos Elastoméricos Utilizados En Disipadores de Energía. Mecánica Comput..

[38] Morita S., Kitagawa K., Ozaki Y. (2009). Hydrogen-Bond Structures in Poly(2-Hydroxyethyl Methacrylate): Infrared Spectroscopy and Quantum Chemical Calculations with Model Compounds. Vib. Spectrosc..

[39] Moses D.N., Harreld J.H., Stucky G.D., Waite J.H. (2006). Melanin and Glycera Jaws: Emerging Dark Side of a Robust Biocomposite Structure. J. Biol. Chem..

[40] Stuart B.H. (2005). Infrared Spectroscopy: Fundamentals and Applications.

[41] White J.L. (1971). Interpretation of Infrared Spectra of Soil Minerals. Soil Sci..

[42] Huang C.W., Sun Y.M., Huang W.F. (1997). Curing Kinetics of the Synthesis of Poly(2-Hydroxyethyl Methacrylate) (PHEMA) with Ethylene Glycol Dimethacrylate (EGDMA) as a Crosslinking Agent. J. Polym. Sci. Part A Polym. Chem..

[43] Singh N.K., Lesser A.J. (2011). A Physical and Mechanical Study of Prestressed Competitive Double Network Thermoplastic Elastomers. Macromolecules.

[44] Morita S., Ye S., Li G., Osawa M. (2004). Effect of Glass Transition Temperature (Tg) on the Absorption of Bisphenol A in Poly(Acrylate)s Thin Films. Vib. Spectrosc..

[45] Choi S.S., Hong J.P., Seo Y.S., Chung S.M., Nah C. (2006). Fabrication and Characterization of Electrospun Polybutadiene Fibers Crosslinked by UV Irradiation. J. Appl. Polym. Sci..

[46] Zalucha D.J., Abbey K.J. (2007). The Chemistry of Structural Adhesives: Epoxy, Urethane, and Acrylic Adhesives. Handbook of Industrial Chemistry and Biotechnology.

[47] De Freitas P.S., Wirz D., Stolz M., Göpfert B., Friederich N.F., Daniels A.U. (2006). Pulsatile Dynamic Stiffness of Cartilage-like Materials and Use of Agarose Gels to Validate Mechanical Methods and Models. J. Biomed. Mater. Res.-Part B Appl. Biomater..

[48] Wan L.Q., Jiang J., Miller D.E., Guo X.E., Mow V.C., Lu H.H. (2011). Matrix Deposition Modulates the Viscoelastic Shear Properties of Hydrogel-Based Cartilage Grafts. Tissue Eng.-Part A.

[49] Buckley C.T., Thorpe S.D., O’Brien F.J., Robinson A.J., Kelly D.J. (2009). The Effect of Concentration, Thermal History and Cell Seeding Density on the Initial Mechanical Properties of Agarose Hydrogels. J. Mech. Behav. Biomed. Mater..

[50] Yu B., Kang S.Y., Akthakul A., Ramadurai N., Pilkenton M., Patel A., Nashat A., Anderson D.G., Sakamoto F.H., Gilchrest B.A. (2016). An Elastic Second Skin. Nat. Mater..

